# Diagnosis of pancreaticobiliary malignancy by detection of minichromosome maintenance protein 5 in bile aspirates

**DOI:** 10.1038/sj.bjc.6604342

**Published:** 2008-04-15

**Authors:** L Ayaru, K Stoeber, G J Webster, A R W Hatfield, A Wollenschlaeger, O Okoturo, M Rashid, G Williams, S P Pereira

**Affiliations:** 1 The Institute of Hepatology, University College London, London, UK; 2 Wolfson Institute for Biomedical Research, University College London, London, UK; 3 Department of Pathology, University College London, London, UK; 4 Department of Gastroenterology, University College London Hospitals NHS Foundation Trust, London, UK

**Keywords:** pancreatic cancer, biliary tract cancer, biliary stricture, MCM

## Abstract

Biliary brush cytology is the standard method of sampling a biliary stricture but has a low sensitivity for the detection of malignancy. We have previously shown that minichromosome maintenance (MCM) replication proteins (Mcm2–7) are markers of dysplasia and have utilised these novel biomarkers of growth for the diagnosis of cervical and bladder cancer. We aimed to determine if MCM proteins are dysregulated in malignant pancreaticobiliary disease and if levels in bile are a sensitive marker of malignancy. In 30 tissue specimens from patients with malignant/benign biliary strictures, we studied Mcm2 and -5 expression by immunohistochemistry. Bile samples were also collected prospectively at endoscopic retrograde cholangiopancreatography from 102 consecutive patients with biliary strictures of established (*n*=42) or indeterminate aetiology (*n*=60). Patients with indeterminate strictures also underwent brush cytology as part of standard practice. Bile sediment Mcm5 levels were analysed using an automated immunofluorometric assay. In benign biliary strictures, Mcm2 and -5 protein expression was confined to the basal epithelial proliferative compartment – in contrast to malignant strictures where expression was seen in all tissue layers. The percentage of nuclei positive for Mcm2 was higher in malignant tissue (median 76.5%, range 42–92%) than in benign tissue (median 5%, range 0–33%) (*P*<0.0005), with similar results for Mcm5. Minichromosome maintenance protein 5 levels in bile were significantly more sensitive than brush cytology (66 *vs* 20%; *P*=0.004) for the detection of malignancy in patients with an indeterminate stricture, with a comparable positive predictive value (97 *vs* 100%; *P*=ns). In this study, we demonstrate that Mcm5 in bile detected by a simple automated test is a more sensitive indicator of pancreaticobiliary malignancy than routine brush cytology.

The diagnosis of pancreatic and biliary tract cancer at an early stage of disease remains difficult and there are currently no established methods of surveillance for biliary tract cancer in patients with primary sclerosing cholangitis (PSC). Current diagnostic modalities include serum tumour markers and imaging but are not specific enough to allow for confident confirmation of malignancy especially in its early stages and therefore cytological/biopsy specimens are usually acquired from biliary strictures/masses (Lee, 2006). Brush cytology is the most commonly used method of sampling a biliary stricture (De Bellis *et al*, 2002a) as it is relatively easy to perform, does not compromise resection margins in potentially resectable cases and has a high specificity (96–100%) for malignancy. However, cytology has a low sensitivity (9–57%) (de Bellis *et al*, 2002b; Baron *et al*, 2004; Harewood *et al*, 2004; Moreno Luna *et al*, 2006) for the detection of malignancy, which is even lower if cells are acquired from bile aspirates (6–32%) (De Bellis *et al*, 2002a). The poor detection rate may stem from a number of factors, including the desmoplastic nature of biliary tract cancers, failure to obtain an adequate cellular yield and morphological changes induced by inflammation and necrosis. Furthermore, to some extent, the interpretation of cytological specimens is subjective and observer-dependent and more accurate quantitative tests would be desirable.

Despite the detection of several molecular genetic alterations in pancreatic and biliary tract cancer, their reported low frequency in biological samples has limited their usefulness as diagnostic markers (Gress, 2004). For example, neither K-Ras nor p53 mutational analysis has been shown to be superior to conventional cytopathology for the diagnosis of pancreaticobiliary tumours (Khan *et al*, 2005).

The initiation of DNA replication represents a final and critical step in growth regulation and lies downstream at the convergence point of growth regulatory pathways (Williams and Stoeber, 1999). Minichromosome maintenance proteins (Mcm2–7) participate in the assembly of prereplicative complexes to establish competence for initiation of DNA synthesis (DNA replication licensing). All six Mcm proteins are essential for replication, are present in all phases of the proliferative cell cycle but are tightly downregulated in the quiescent, terminally differentiated and senescent ‘out-of-cycle’ states. The presence of one protein reflects the presence of the other five as all six are loaded together onto DNA as a heterohexamer on exit from metaphase (Blow and Hodgson, 2002). We have shown that these biomarkers detect, in addition to actively proliferating cells, cells with growth potential (Stoeber *et al*, 2001). We have also shown that dysregulation of MCM proteins is an early event in epithelial carcinogenesis, which occurs in a wide range of preneoplastic and neoplastic states (Freeman *et al*, 1999) resulting in exfoliation of MCM-positive tumour cells. Moreover, we have utilised these novel biomarkers of growth as diagnostic markers of cervical, genitourinary tract and oesophageal cancer (Williams *et al*, 1998, 2004; Stoeber *et al*, 1999, 2002).

On the basis of these data in other solid-organ tumours, we proposed that detection of MCM proteins in exfoliated tumour cells might be a potentially sensitive indicator of pancreaticobiliary neoplasia. Here we describe a novel automated liquid-phase immunofluorometric assay to quantify Mcm5 levels in biliary aspirates obtained from patients undergoing endoscopic retrograde cholangiopancreatography (ERCP) for the diagnosis and treatment of biliary strictures.

## METHODS

### Patients

Between 2004 and 2006, 113 consecutive patients were invited to participate in the study for evaluation of indeterminate, or established, benign and malignant biliary tract strictures. Seven patients were excluded due to inability to sedate the patient adequately (*n*=3), bile not aspirated (*n*=2), ampulla not identified (*n*=1) or patient did not agree to participate in the study (*n*=1). A total of 106 patients underwent ERCP with aspiration of bile for Mcm5 analysis and parallel biliary brush cytology obtained as part of routine diagnostic practice in indeterminate strictures. Four patients were excluded after bile collection as we did not have access to sufficient follow-up information to be confident of a diagnosis. Therefore, a total of 102 patients were included in the study. A diagnosis of malignancy was made by positive cytology/biopsy or evidence of disease progression on imaging. Benign disease was established by negative pathology and a median of 39 (range 21–48) months clinical follow-up. The study was approved by the Joint UCLH/UCL ethical committee and all patients gave written informed consent.

### Brush cytology of biliary strictures

If a stricture of indeterminate aetiology was present at ERCP, biliary brush cytology was collected by standard technique. A wire-guided sheathed cytology brush (Combocath, Microinvasive; Boston Scientific, Notick, MA, USA) was advanced across the stricture several times before being resheathed and the sheathed brush withdrawn from the endoscope. The cytology specimen was then transferred immediately to glass slides by smearing the cellular material from the brush directly onto two slides. These were fixed and later stained for malignant cells using the standard Papanicolaou technique for smears. Brush cytology samples were analysed by expert cytopathologists within the context of a multidisciplinary cancer review meeting. Cytology was classified as malignant or no definitive evidence of malignancy (highly suspicious, low-grade dysplasia, inflammatory, normal).

### Immunohistochemistry

Formalin-fixed archival blocks were selected from the files at University College London Hospital. Sections (4 *μ*m) were cut using a sledge microtome and placed onto Superfrost Plus slides. After drying the slides overnight at 60°C, sections were deparaffinised in xylene and rehydrated in water. Antigen retrieval was performed by pressure cooking sections in 0.1 mmol l^−1^ citrate buffer (pH 6.0) at 15 p.s.i. (103.4 kPa) for 2 min.

Tissue sections cut on to Superfrost Plus slides were stained manually or using a standard protocol, as described below. Following antigen retrieval, the slides were washed thrice (using Tris buffered saline with 0.1% Tween 20 for this and subsequent washes). Endogenous peroxidase activity was quenched with peroxidase-blocking solution (DAKO, Ely, UK) for 15 min. Sections were incubated with primary antibody for 45 min. Mouse anti-human monoclonal Mcm2 and rabbit anti-human polyclonal Mcm5 antibodies were obtained from BD Transduction Laboratories (Lexington, KY, USA). The slides were incubated with the secondary antibody for 2 h and developed with 3,3-diaminobenzidine for 10 min. Slides were then counterstained with Mayer's haematoxylin, differentiated in 1% acid alcohol, dehydrated and cleared in xylene. Coverslips were applied with Leica CV Mount (Leica, Nussloch, Germany). Incubation without the primary antibody was used as a negative control and colonic epithelial sections were used as positive controls. Those sections mounted on DAKO ChemMate capillary gap slides were stained using the DAKO TechMate 500 immunostainer (DAKO, Cambridge, UK). Microscopic images were acquired with an Olympus BX51 light microscope/CCD camera setup and ANAlysis image-capturing software (Soft Imaging Systems GmbH, Munster, Germany). A semiquantitative determination of the extent of staining was obtained by calculating a labelling index for each protein stained. At least 200 epithelial nuclei were assessed per case. Results were expressed as a percentage of positively stained nuclei out of the total number of nuclei counted in representative microscopic fields. The median and range of labelling indices were calculated.

### Bile aspirate collection and storage

After biliary brushing, 5–10 ml of bile was aspirated from directly above the stricture via a standard ERCP catheter. Storage buffer (10 × phosphate-buffered saline (PBS), 5% bovine serum albumin, 1 M sucrose, 0.2% NaN_3_) containing one complete mini EDTA-free protease inhibitor cocktail tablet (Roche Diagnostics Ltd, Lewes, East Sussex, UK) per 10 ml of buffer was added to bile aspirates at one-tenth aspirate volume and mixed with the sample. Bile aspirates in storage buffer were transferred into 15 ml cryovials, placed in dry ice and stored at −80°C within 6 h.

### Processing of standards and bile aspirates

Aspirates were analysed in a blinded manner for immunofluorometric Mcm5 detection. Standards for the immunofluorometric Mcm5 assay were prepared by serial dilution of lysates from asynchronous HeLa S3 cultures to 1500, 5000, 15 000, 50 000 and 150 000 cells per well. Standards and bile samples were processed as described previously (Stoeber *et al*, 2002). Briefly, standards and clinical samples were thawed, and the cells were isolated by centrifugation at 1500 **g** for 5 min at 4°C. The supernatants were discarded, and the cell pellets were washed three times with 500 *μ*l of PBS. Cell pellets were resuspended in 250 *μ*l of processing buffer (PBS, 0.4% sodium dodecyl sulphate (SDS), 0.02% NaN_3_). Cell lysates were prepared by incubating the resuspended samples at 95°C for 45 min. The DNA in each sample was sheared by passing the lysates through a 21-gauge needle (Terumo Europe NV, Leuven, Belgium), and nucleic acids were digested with DNase I (20 U ml^−1^; Roche Diagnostics) and RNase A (1 mg ml^−1^; Sigma-Aldrich UK Ltd, Dorset, UK) for 2 h at 37°C. The samples were centrifuged at 15 000 **g** for 10 min to pellet the cell debris, the supernatants were collected and 50 *μ*l of each was directly used in the immunofluorometric assay.

### Automated immunofluorometric measurement of Mcm5 levels in bile aspirates

Monoclonal antibodies (MAbs) 12A7 and 4B4 raised against His-tagged human Mcm5 were protein A-purified from hybridoma supernatants as described previously (Stoeber *et al*, 2002). Protein A-purified MAb 4B4 was labelled with europium using a DELFIA Eu-labelling kit (Perkin-Elmer Life Science, Wallac Oy, Turku, Finland) according to the manufacturer's instructions. The assay was standardised using HeLa cells as described above and previously (Stoeber *et al*, 2002), and one fluorescence unit was defined as the signal generated by the Mcm5 contents of one proliferating HeLa S3 cell, approximately 10^5^ Mcm5 molecules (Kearsey and Labib, 1998). DELFIA research reagents were obtained from Perkin-Elmer Life Science. Multibuffer was prepared from 0.2 vol 5 × DELFIA assay buffer (Perkin-Elmer), 0.125 vol DELFIA TSH-Ultra assay buffer (Perkin-Elmer) and 0.1 vol Tween 20 (Sigma). All other reagents were obtained from Sigma-Aldrich. Immunofluorometric measurements of Mcm5 levels were performed as described previously (Stoeber *et al*, 2002). Standard curves were constructed from fluorescence values generated by the blank and standard wells, and the fluorescence values of the bile aspirate samples were calculated with the Multicalc Advanced Immunoassay Data Management package (Perkin-Elmer Life Science). The reliability of the test was maintained by assaying the Mcm5 content of a known number of HeLa cells as an internal standard when bile samples were assayed.

For immunofluorometric measurement of Mcm5 levels, assay standards, control samples and bile aspirate samples were run as duplicates and the mean of the duplicate results reported. For acceptance of immunofluorometric measurements in the assay, the following coefficients of variations were required: CV<20% for results between 1500 and 5000 cells per well standard curve points; CV<15% for results between 5000 and 15 000 cells per well; and CV<10% for results >15 000 cells per well. On completion of the study, patient data were decoded and the immunofluorometric signals compared with biliary brushing results.

### Immunoassay performance

In our analysis, we used 1000 cells per well as the lower detection limit because the within-batch coefficient of variation of the assay was less than 25% in all samples with cell dilutions above 1000 cells per well, but in only one-quarter of samples below this limit. Samples that generated a fluorescence signal below that corresponding to 1000 cells per well were reported as having fewer than 1000 cells per well.

### Statistical analysis

Sensitivity and specificity characteristics of the immunofluorometric Mcm5 test for the detection of pancreaticobiliary malignancy were presented as a receiver operating characteristics (ROC) curve. The area under the nonparametric ROC curve was used to assess the overall accuracy of the test. Two cut points were used to demonstrate test performance under different circumstances as follows: (i) at the lower detection limit of the assay (i.e. 1000 cells per well), where sensitivity of the test was maximal and (ii) where specificity was 100% (i.e. 1780 cells per well). An exact 95% confidence interval (CI) for each proportion, including sensitivity, specificity and predictive values of Mcm5 and cytology, was derived assuming a binomial distribution using Graph Pad Prism 4 (Graph Pad Software, Inc., San Diego, CA, USA) and/or SPSS software, version 11.5 (SPSS Inc., Chicago, IL, USA).

The sensitivity determined for biliary brush cytology was compared with that of the immunofluorometric Mcm5 test using McNemar's test for paired proportions. The level of the signal was compared between patient groups using the Mann–Whitney *U*-test. All statistical tests were two-tailed, and a 5% level was used to indicate statistical significance.

## RESULTS

### MCM proteins are dysregulated in malignant pancreaticobiliary disease

**Figure 1:**
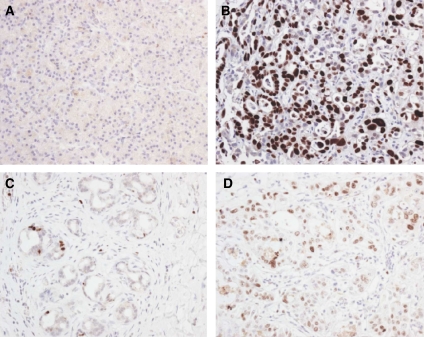
Minichromosome maintenance protein 2 expression in benign and neoplastic pancreaticobiliary diseases. (**A**) Normal pancreas showing absence of Mcm2 expression. (**B**) Moderately to poorly differentiated pancreatic adenocarcinoma showing high levels of Mcm2 expression. Occasional viable Mcm2-negative cells are present. (**C**) Section of a benign hilar stricture showing occasional Mcm2-positive cells at the base of glands. (**D**) Moderately to poorly differentiated cholangiocarcinoma showing high levels of Mcm2 expression. Mcm2=minichromosome maintenance protein 2.

The pattern of Mcm2 and -5 protein expression was assessed by immunohistochemistry in morphologically normal, benign and malignant biliary and pancreatic tissue derived from biopsies of masses associated with biliary strictures (Table 1) (Figure 1A–D).

**Figure 2:**
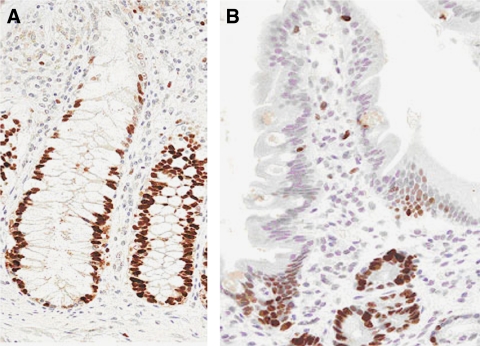
Immunohistochemistry for Mcm5 in the (**A**) colonic crypt and (**B**) ampulla. At the base of epithelium, nuclei of epithelial cells are positive for Mcm5 (dark brown) in contrast to surface of epithelium where cells do not express Mcm5. Mcm5=minichromosome maintenance protein 5.

As a positive control for experiments on pancreaticobiliary tissues, the pattern of Mcm2 and -5 protein expression was assessed in the colonic crypt as we have described previously (Stoeber *et al*, 2001). There was nuclear staining of 70 and 74% of cells in the lower third of colonic crypts for Mcm2 and Mcm5 respectively, and less than 5% in the upper third (Figure 2A). In the normal ampulla, which has glands similar to the colon, the expression of MCM proteins was limited to the basal proliferative epithelial compartment (Figure 2B).

**Figure 3:**
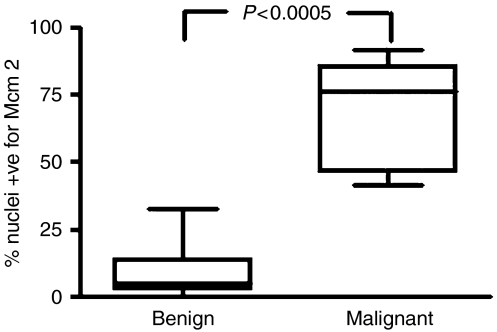
Box-and-whisker plot of range 25th–75th percentile and median Mcm2 expression in benign and malignant biliary strictures. Mcm2=minichromosome maintenance protein 2.

The expression of Mcm2 and Mcm5 (not shown) was extremely low in normal pancreas and bile duct (<5% positively stained nuclei), in keeping with previous observations that reduced proliferative capacity in stable tissues (e.g., liver and thyroid) is coupled to repression of origin licensing through downregulation of MCM helicase subunits (Stoeber *et al*, 2001). In contrast, in pancreatic cancer, ampullary carcinoma and cholangiocarcinoma, high levels of Mcm2 and -5 expression were seen in all tissue layers indicative of cell-cycle re-entry (Stoeber *et al*, 1998, 2001; Wharton *et al*, 2001). The percentage of nuclei positive for Mcm2 was higher in malignant tissue (median 76.5%, range 42–92%) than that in benign tissue (median 5%, range 0–33%) (*P*<0.0005) (Figure 3). Similarly, the percentage of nuclei positive for Mcm5 was higher in malignant strictures (median 91%, range 84–95%, *n*=5) than in benign strictures (median 4%, range 3–8%, *n*=5) (not shown).

### Mcm5 in bile aspirates compared with routine brush cytology to diagnose pancreaticobiliary malignancy

Bile aspirates were acquired from 102 patients with biliary strictures of established (*n*=42) or indeterminate aetiology (*n*=60) and the final diagnoses are shown in Table 2. The median age of the patients was 67 years (33–103 years; M:F 1 : 1). A final diagnosis of malignancy was eventually made in 44/60 patients with indeterminate strictures established by brush cytology (*n*=9), endoscopic ultrasound-fine-needle aspiration (EUS-FNA) (*n*=1), intraductal biopsy (*n*=7), percutaneous biopsy (*n*=16), resection specimens (*n*=3) or clinical course (*n*=8). Two brush cytology specimens were highly suspicious for malignancy and a final diagnosis of cancer was made in both. First (i) endoscopic biopsy performed at the time of brush cytology (*n*=20), (ii) percutaneous biopsy (*n*=9) or (iii) EUS-FNA (*n*=1) performed on a separate occasion was obtained in 30/44 patients with eventual diagnoses of malignant strictures and was positive for cancer in 13/30 (43%). Fourteen patients with final diagnoses of malignant strictures underwent a second biopsy/EUS-FNA, which was positive for cancer in 11/14 (79%). Twenty-four out of forty-four (55%) patients underwent at least two separate attempts (range 1–3) at tissue acquisition before malignancy was confirmed. Benign disease was confirmed by negative pathology during a mean of 36 (range 21–48) months follow-up.

**Figure 4:**
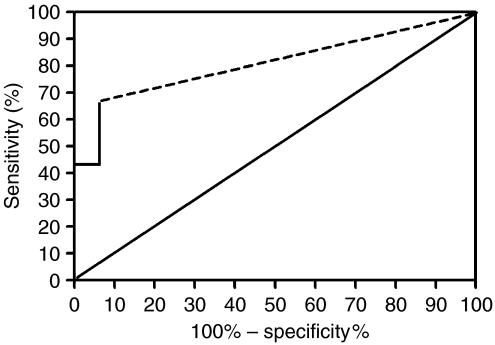
Receiver operating characteristic curve of immunofluorometric Mcm5 test. The jagged curve (solid line) is the nonparametric ROC curve. The diagonal line is the reference line. Area under the curve is 80% (95% CI 70–91). Mcm5=minichromosome maintenance protein 5; ROC=receiver operating characteristic.

The performance of the immunofluorometric Mcm5 assay as a diagnostic test for pancreaticobiliary malignancy in patients with indeterminate strictures is shown as an ROC curve (Figure 4). The test discriminated with high accuracy between patients with and without malignancy, as demonstrated by an area under the ROC curve of 0.80 (95% CI 0.70–0.91), which was significantly larger than the area predicted by the null hypothesis (0.5) (*P*<0.0004). In other words, a randomly selected patient with pancreaticobiliary malignancy would have an 80% probability of having an immunofluorometric Mcm5 value that is greater than that for a randomly selected patient without malignancy. Three patients with malignant strictures had bile samples aspirated on two separate occasions and the mean fluorescence differed by <5%.

Evaluation of the test in comparison with brush cytology in the 60 patients with indeterminate strictures at the time of sample collection is shown in Table 3 at two different performance levels: (i) 1000 cells per well (lower detection limit of the assay) and (ii) 1780 cells per well (high specificity). At the 1000-cell cut point, the test had 66% (29/44) sensitivity and 97% (29/30) positive predictive value. The Mcm5 test detected 20/44 (45%) additional cases of cancer that were not detected by brush cytology. At the 1780 cut point, the test had 43% sensitivity and 100% positive predictive value. The sensitivity and specificity of the Mcm5 test at the 1000-cell cut point in strictures with established diagnoses were 62 and 92%, respectively. In patients with a final diagnosis of a benign stricture, 3/39 (gallstones *n*=1, PSC *n*=2) had biliary Mcm5 values greater than 1000 cells per well.

The performance of the immunofluorometric Mcm5 test according to selected final diagnoses is shown in Table 4. The Mcm5 immunofluorometric signal for patients with bile duct stones with or without cholangitis (median<1000) was not higher than that for other patients negative for malignancy, where the median signal was also below the lower detection limit of 1000 cells per well (Mann–Whitney *U*-test, *P*=0.78). Significant differences were detected between those samples from patients with cholangiocarcinoma (median 1070, *P*=0.03) or pancreatic cancer (median 1490, *P*=0.003) and those with inflammatory strictures (median<1000). The level of signal was not significantly different (*P*=0.16) between cholangiocarcinoma and pancreatic cancer.

## DISCUSSION

In patients who present with a pancreaticobiliary stricture of indeterminate origin, biliary brush cytology is the most commonly used invasive technique to distinguish benign from malignant disease. The technique offers the clinician almost definitive diagnostic certainty when positive for malignancy (specificity 96–100%) but has a poor ability to detect malignancy (sensitivity 18–57%) (de Bellis *et al*, 2002b; Baron *et al*, 2004; Harewood *et al*, 2004; Moreno Luna *et al*, 2006).

This proof of principle study has shown that the sensitivity of the automated immunofluorometric Mcm5 test on bile aspirates for detecting pancreaticobiliary malignancy was superior (four times more at the 1000 cells per well cut point) to that of brush cytology, while maintaining a high specificity. The high positive predictive value for malignancy of this test in this group of patients is of particular importance as a positive test would allow the clinician to make treatment recommendations with a high degree of certainty. We have previously shown that the Mcm5 immunofluorometric test is an accurate test for bladder, prostate and oesophageal cancer (Stoeber *et al*, 2002; Williams *et al*, 2004), and here we show its utility for the diagnosis of pancreatic and biliary tract cancer. Taken together, these studies indicate that the Mcm5 immunofluorometric assay is a robust test to detect exfoliated malignant cells in body fluids. Moreover, as these malignancies are associated with different sets of genetic mutations leading to uncontrolled cell proliferation, this study provides further evidence supporting the hypothesis that the convergence point of growth regulatory pathways that control cell proliferation is the initiation of genome replication in which the MCM complex plays an essential part.

The median level of expression of Mcm5 in malignant bile samples was lower than that we detected in urine and oesophageal aspirates obtained from patients with bladder and oesophageal cancer respectively (Stoeber *et al*, 2002; Williams *et al*, 2004). The absolute values obtained are however not the most important factor in determining the usefulness of the test but whether a cutoff point can be obtained that helps to distinguish between benign and malignant bile duct strictures by a clinically important margin, which this test did in the population studied.

Importantly, inflammatory strictures secondary to PSC and chronic pancreatitis, which are particularly difficult to distinguish from malignant disease with conventional imaging (Lazaridis and Gores, 2005; Hamer and Feuerbach, 2006), had median biliary Mcm5 values below the detection limit of the assay. Benign/normal tissues did express MCM proteins at low levels (median 5% of cells stained by immunohistochemistry), but as the cells are located in the basal epithelium they were probably not exfoliated in bile in large numbers and therefore not detected by the immunofluorometric test. Interestingly, patients with bile duct stones and cholangitis – who might be expected to have ulceration and exposure of the stem-transit compartment of biliary epithelium to bile – also had a median Mcm5 level in bile below the detection limit of the assay, reflecting low shedding of reactive Mcm5-positive cells. This was in contrast to our previous data in patients with renal calculi and oesophageal ulceration, where the test detected positive cells in urine and luminal secretions, though of a magnitude below that of patients with urothelial or oesophageal carcinoma (Stoeber *et al*, 2002; Williams *et al*, 2004).

The immunofluorometric Mcm5 level results in bile were corroborated by immunohistochemistry data, which showed that the percentage of nuclei positive for Mcm2 and -5 was higher in malignant strictures than benign strictures. To date, we have performed immunohistochemistry on more samples using anti-Mcm2 than anti-Mcm5 antibodies. The MCM complex consists of six proteins, all of which are necessary to support initiation of replication. By detecting the presence of one protein, one can infer the presence of the other five. However, to verify this we tested 10 cases for both Mcm5 and Mcm2, which showed a similar % of nuclei positive for MCM proteins.

The molecular diagnosis of pancreaticobiliary malignancies has been the subject of intensive investigation (Gress, 2004), but to date few such tests have been developed and incorporated into routine clinical practice. A recent study of fluorescence *in situ* hybridisation to detect chromosomal abnormalities in biliary brush cytology samples demonstrated results comparable to our study with a sensitivity of 59–70% and specificity of 86–100% for the diagnosis of pancreaticobiliary malignancy compared with a sensitivity of 4–20% for conventional brush cytology (Moreno Luna *et al*, 2006).

The sensitivity of biliary brush cytology (20%) in our study is at the lower end of the range in the published literature although other published studies (Baron *et al*, 2004; Harewood *et al*, 2004; Moreno Luna *et al*, 2006) have reported similar sensitivities of around 20%. One possible explanation is that patients referred to our centre had small volume, difficult to diagnose tumours, as demonstrated by the fact that the majority of the first cytology/biopsy episodes of malignant strictures were negative for cancer. Bile duct forceps biopsy (Jailwala *et al*, 2000), EUS-guided FNA of strictures (Fritscher-Ravens *et al*, 2004) or a cytopathologist within the endoscopy room to immediately analyse samples (De Bellis *et al*, 2002a) can all independently add to the detection rate of brush cytology. However, intraductal biopsy, which often requires a biliary sphincterotomy (Ponchon *et al*, 1995), and EUS-guided FNA of strictures are technically challenging procedures with a higher risk of complications than brush cytology and therefore are not usual practice in our unit or at other centres (Lee, 2006) when acquiring tissue for the first time. The advantage of the immunofluorometric test is that it is based on bile aspirates, which is technically easier to acquire than all of the methods mentioned.

The sensitivity of the Mcm5 test depends in part on the cellularity of bile, which can be acellular in up to 30% of samples (Mansfield *et al*, 1997), so that the sensitivity of bile aspirate Mcm5 cannot be expected to reach 100%. In contrast, the cellularity of brush cytology is much greater than bile aspirates and we are currently assessing whether cells derived from brush cytology will further improve the sensitivity of the Mcm5 test. As all cancer tissues studied expressed MCM proteins, a sensitivity approaching 100% with cells derived from brush cytology applied to the immunofluorometric Mcm5 test is theoretically achievable. Future work will aim to replicate these results in a new cohort of patients and also examine the utility of this test in distinguishing PSC from cholangiocarcinoma and chronic pancreatitis from pancreatic cancer in a larger number of patients.

In conclusion, we have demonstrated that immunofluorometric detection of Mcm5 in bile aspirates is a sensitive and specific diagnostic test for pancreaticobiliary malignancy. The test detects a variety of cancer types including those often missed by biliary brush cytology. This simple method for detecting pancreaticobiliary malignancy is now automated allowing for easy translation into a clinical diagnostic test.

## Figures and Tables

**Table 1:** Mcm2 expression in masses associated with biliary strictures

**Tissue**	**Cases (*n*=30)**	**Mcm2 % (range)**
Normal pancreas	4	3.5 (0–9.9)
Inflammatory biliary epithelium	5	6 (0–16)
Benign ampulla^2^	3	26 (16–38)
Chronic pancreatitis	3	5.5 (0–14)
Pancreatic cancer	5	80 (30–91)
Ampullary cancer	5	56 (40–75)
Cholangiocarcinoma	5	86 (80–92)

Abbreviation: Mcm2=minichromosome maintenance protein 2.

2 Normal ampulla *n*=1, ampulla with chronic inflammation *n*=1, villous adenoma with low-grade dysplasia *n*=1.

**Table 2:** Final diagnosis of bile duct strictures in study of Mcm5 test in bile aspirates

**Stricture**	**Established diagnoses (*n*=42)**	**Final diagnoses of indeterminate strictures (*n*=60)**	**Total (*n*=102)**
*Malignant*	18	44	62
Cholangiocarcinoma	12	15	27
Pancreatic cancer	4	19	23
Ampullary carcinoma	0	4	4
Mucinous tumours	2	2	3
Hepatocellular carcinoma	0	1	1
Lymphoma	0	1	1
Metastases to bile duct	0	1	1
Neuroendocrine tumour	0	1	1
			
*Benign*	24	16	40
Gallstones	9	5	14
Primary sclerosing cholangitis	6	3	9
Chronic pancreatitis	3	2	5
Autoimmune pancreatitis	0	3	3
Idiopathic	3	1	4
Postoperative	2	1	3
Papillary stenosis	0	0	1
Ampullary adenoma	0	1	1

Abbreviation: Mcm5=minichromosome maintenance protein 5.

**Table 3:** Comparison of the Mcm5 immunofluorometric assay in bile aspirates with brush cytology for the diagnosis of pancreaticobiliary malignancy

	**Sensitivity (95% CI)**	**Specificity (95% CI)**	**PPV (95% CI)**	**NPV (95% CI)**	**Area under curve (95% CI)**
Mcm5 cutoff point >1000	66% (50–79)^2^	94% (70–100)^3^	97% (90–100)	50% (32–68)	80% (70–91)
Mcm5 cutoff point >1780	43% (29–57)	100%	100%	39 % (24–53)	72% (58–85%)
Brush cytology	20% (7–35)^2^	100%^3^	100%	31% (18–44)	60% (40–76)

Abbreviations: Mcm5=minichromosome maintenance protein 5; NPV=negative predictive value; PPV=positive predictive value.

2 *P*=0.004 for sensitivity Mcm5 *vs* cytology

3 *P*=not significant.

**Table 4:** Immunofluorometric Mcm5 test performance in patient groups

**Patient group**	**Group size (*N*)**	**Median signal**	**Interquartile range**
*Negative for cancer*	40	<1000	<1000–<1000
Gallstones	15	<1000	<1000–<1000
Chronic inflammation^2^	14	<1000	<1000–<1000
			
*All cancers*	62	1198	<1000–3495
Cholangiocarcinoma	27	1070	<1000–2060
Pancreatic cancer	23	1490	<1000–5340

Abbreviation: Mcm5=minichromosome maintenance protein 5.

2 Primary sclerosing cholangitis and chronic pancreatitis.
